# Cultivar and Metal-Specific Effects of Endophytic Bacteria in *Helianthus tuberosus* Exposed to Cd and Zn

**DOI:** 10.3390/ijms18102026

**Published:** 2017-09-21

**Authors:** Blanca Montalbán, Sofie Thijs, Mª Carmen Lobo, Nele Weyens, Marcel Ameloot, Jaco Vangronsveld, Araceli Pérez-Sanz

**Affiliations:** 1 Departamento de Investigación Agroambiental, IMIDRA, Finca “El Encín”, Autovía del Noreste A-2 Km 38.2, 28800 Alcalá de Henares, Madrid, Spain; carmen.lobo@madrid.org (Mª.C.L.); a.perez@nhm.ac.uk (A.P.-S.); 2 Environmental Biology, Centre for Environmental Sciences, Hasselt University, Agoralaan Building D, BE3590 Diepenbeek, Belgium; sofie.thijs@uhasselt.be (S.T.); nele.weyens@uhasselt.be (N.W.); jaco.vangronsveld@uhasselt.be (J.V.); 3 Biomedical Research Department, Hasselt University, Agoralaan building D, BE3590 Diepenbeek, Belgium; marcel.ameloot@uhasselt.be; 4 Department of Earth Sciences, Natural History Museum, Cromwell Road, London SW7 5BD, UK

**Keywords:** metal contaminated soil, *Helianthus tuberosus*, phytoremediation, high biomass crop, green fluorescent protein, plant growth promoting bacteria

## Abstract

Plant growth promoting endophytic bacteria (PGPB) isolated from *Brassica napus* were inoculated in two cultivars of *Helianthus tuberosus* (VR and D19) growing on sand supplemented with 0.1 mM Cd or 1 mM Zn. Plant growth, concentrations of metals and thiobarbituric acid (TBA) reactive compounds were determined. Colonization of roots of *H. tuberosus* D19 by *Pseudomonas* sp. 262 was evaluated using confocal laser scanning microscopy. *Pseudomonas* sp. 228, *Serratia* sp. 246 and *Pseudomonas* sp. 262 significantly enhanced growth of *H. tuberosus* D19 exposed to Cd or Zn. *Pseudomonas* sp. 228 significantly increased Cd concentrations in roots. *Serratia* sp. 246, and *Pseudomonas* sp. 256 and 228 resulted in significantly decreased contents of TBA reactive compounds in roots of Zn exposed D19 plants. Growth improvement and decrease of metal-induced stress were more pronounced in D19 than in VR. *Pseudomonas* sp. 262-green fluorescent protein (GFP) colonized the root epidermis/exodermis and also inside root hairs, indicating that an endophytic interaction was established. *H. tuberosus* D19 inoculated with *Pseudomonas* sp. 228, *Serratia* sp. 246 and *Pseudomonas* sp. 262 holds promise for sustainable biomass production in combination with phytoremediation on Cd and Zn contaminated soils.

## 1. Introduction

During the last two decades, the potential use of plants to remediate metal contaminated soils has been intensively investigated. For application of phytotechnologies on metal contaminated soils, and especially in the case of phytoextraction, metal availability, uptake and phytotoxicity are the main limiting factors [1,2,3,4,5]. The interactions between plants and beneficial bacteria may increase the efficiency of phytoextraction because of increased biomass, metal uptake and plant tolerance to toxic metals [6,7,8,9]. Plant growth can be enhanced: (1) indirectly, by preventing growth and activity of plant pathogens through the production of antibiotics or through competition for space and nutrients [10]; and (2) directly, by increasing available nutrients through different mechanisms such as nitrogen fixation [11], solubilization of minerals such as phosphorous and iron [12,13], and production of phytohormones (as IAA, indole-3-acetic acid) [14] and 1-aminocyclopropane-1-carboxylate (ACC) deaminase [15,16]. Metal and nutrient availability can be enhanced by excreting organic acids that decrease pH in the rhizosphere or by enhancing the Fe(III) mobility and other cations through production of siderophores [9,17,18]. Some microorganisms are equipped with metal-resistance/sequestration systems that can contribute to metal detoxification [19]. Plant-associated bacteria can also adsorb metals by binding them to anionic functional groups or to extracellular polymeric substances of the cell wall [20,21,22]. This leads to a reduced metal uptake and translocation inside the plant, improving its growth through decreasing phytotoxicity [8,23]. In a previous study, we showed that bacterial strains isolated from a Zn contaminated soil increased root length of *Brassica napus* seedlings in the presence of Cd and Zn under in vitro conditions [24].

Many studies have evaluated the interactions between plants and their associated bacteria for the removal or stabilization of metals in contaminated soils [25]. In some cases, bacteria isolated from metal tolerant plants promoted the growth of plants from different taxonomic groups [23,26,27,28] and demonstrated high levels of colonization in plant species different from the original host. Several studies have been performed under hydroponic conditions to evaluate the effects of bacteria on growth, metal uptake and production of thiobarbituric acid (TBA) reactive compounds for different plants and metals [29,30,31]. However, most studies tested the use of single inocula on the same plant species. In the light of bacteria-stimulated phytoremediation, it is however important to assess the bacterial colonization of more than one plant cultivar under different metal pollution contexts.

The strategy of bacterial inoculation is one of the most critical steps in phytotechnology applications [32]. The colonization must be effective in order to achieve beneficial effects on plant growth and metal uptake [33]. A profound knowledge about plant growth promoting endophytic bacteria (PGPB) colonization routes and plant–bacteria interactions is essential to develop an effective method of inoculation [34]. The use of fluorescent proteins in non-invasive microscopy is a well-established and valuable tool in biology and biotechnology [35]. Labeling with enhanced green fluorescent protein (EGFP) can be adopted to observe the colonization patterns of bacteria [36,37,38]. GFP has been described to be a good marker for studying bacterial behavior in the rhizosphere and the endosphere [39,40]. Recently, Ma et al. [3] pointed out that endophytes could be a more reliable source of natural biocenosis than rhizobacteria because of their intimate association with plants, although their effects in phytotechnologies still should be investigated more in depth.

*Helianthus tuberosus* L. (Asteraceae) is a high biomass crop used for bio-ethanol production. It is vegetatively propagated by tubers [41] with low production costs and negligible pests and disease problems [42,43]. Several studies have demonstrated the tolerance of this crop to metals such as Cd, Pb and Zn [44,45,46,47,48]. All these characteristics make *H. tuberosus* a promising candidate for phytoremediation of metal contaminated soils, as well as to produce renewable energy. Therefore, the aim of this work was to evaluate the effects of PGPB strains, isolated from *B. napus* growing on a metal contaminated soil, on growth, metal uptake and TBA reactive compounds, in two cultivars of *H. tuberosus* (VR and D19) exposed to Cd and Zn. Of one particular interesting endophytic strain, *Pseudomonas* sp. 262, the colonization of the roots of *H. tuberosus* was studied using confocal laser scanning microscopy.

## 2. Results and Discussion

### 2.1. Plant Growth and Metal Uptake

**Figure 1:**
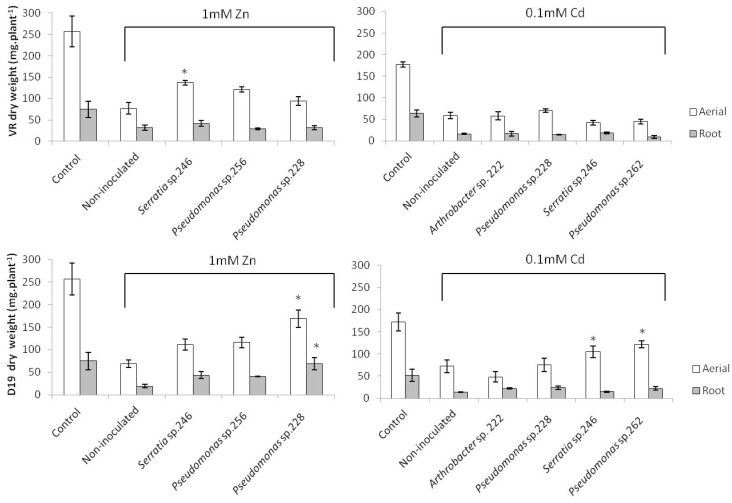
Dry weight (mg·plant^−1^) of the *H. tuberosus* cultivars VR and D19 after three weeks of growth in presence of 1 mM Zn or 0.1 mM Cd. * Significant differences between inoculated and non-inoculated after Tukey’s test, *p* < 0.05; mean values ± SE; *n* = 4.

Exposure to Cd and Zn significantly decreased the weight of *H. tuberosus* in comparison to the non-exposed control plants (Figure 1). In particular, the shoot weight decreased by 57% and the root weight by 67% when plants were exposed to 0.1 mM Cd; the reductions reached 70% and 50% in shoot and root weights in the case plants were grown in presence of 1 mM Zn. Some of the inoculated bacterial strains significantly improved growth of metal exposed plants. In the presence of Zn, inoculation of *Pseudomonas* sp. 228 significantly increased both shoot and root weights of the D19 cultivar, by 145% and 263% respectively (Figure 1). *Serratia* sp. 246 increased the shoot weight of the VR cultivar under Zn exposure by 78%. In Cd exposed plants of the D19 cultivar, inoculation of *Pseudomonas* sp. 262 and *Serratia* sp. 246 significantly increased the shoot weight by 68% and 46%, respectively. These beneficial effects on weight are in line with earlier studies in which positive effects of inoculation with PGPB on growth of plants exposed to metals were reported [49,50,51]. However, these positive effects of the endophytes *Pseudomonas* sp. and *Serratia* sp. have been not described before in a tuberous plant exposed to metals.

In vitro, the inoculated bacterial strains demonstrated plant growth-promoting characteristics like production of IAA, acetoin and ACC deaminase activity that can improve the growth of their host plant (Table 1). Production of IAA and acetoin can stimulate root formation [52,53], and thereby increase the nutrient absorption capacity of the plant. ACC deaminase activity can reduce the ethylene levels generated due to stress, improving the growth of plants in presence of toxic concentrations of metals [16]. It is important to mention that the endophytic bacterial strains that increased the growth of *H. tuberosus* also increased the length of the roots of *Brassica napus* seedlings in vertical agar plates containing toxic concentrations of Cd and Zn [24]. This suggests that these endophytes are beneficial for plants from different families.

The bacterial inoculation also affected the metal concentrations in both cultivars of *H. tuberosus* (Table 2). The Zn concentration significantly decreased in roots of the VR cultivar inoculated with *Pseudomonas* sp. 228. No significant differences were found between inoculated and non-inoculated plants of the D19 cultivar. In the case of Cd exposure, the effects were different. Inoculation of *Pseudomonas* sp. 228 significantly increased the Cd concentration in roots of the D19 cultivar in comparison to non-inoculated plants. In contrast, inoculation of *Pseudomonas* sp. 262 and *Arthrobacter* sp. 222 decreased the Cd concentration in roots of the VR cultivar. *Serratia* sp. 246 and *Pseudomonas* sp. 262 also decreased the concentrations of Cd in the shoots of, respectively, the VR and the D19 cultivar. In metal contaminated nutrient solutions, metals are almost entirely available to plants. Therefore, the effects of the bacteria on the plant uptake could be masked because of the high metal uptake that usually occurs in these cases. Wan et al. [30] did not observe significant differences in Cd uptake by hydroponically grown *Solanum nigrum* after inoculation of *Serratia nematodiphila* LRE07 in the presence of high Cd concentrations. These authors concluded that the effect of the strain was more significant at lower concentrations (10 µM of Cd). Moreover, the decreases in Cd and Zn concentrations in inoculated plants could be due to the capacity of some bacteria to adsorb and immobilize toxic ions from the solution through the production of extracellular polysaccharides and proteins that can bind and precipitate metals [54]. It this way, bacteria can reduce the phytotoxic effects of the metals improving the growth of the host plant [21,23,29]. Several authors have reported such effect in different plant species and diverse growth conditions. Marques et al. [55] observed that the Cd and Zn concentrations in roots of *Helianthus annuus* decreased after inoculation with *Chrysiobacterium humi*, isolated from a Cd-Zn contaminated soil. They attributed this effect to the fact that some bacteria can share the metal load with the plant, thereby decreasing the metal uptake in the plant. Tripathi et al. [56] described that the growth of *Phaseolus vulgaris* improved after inoculation of *Pseudomonas putida* KNP9 in a soil spiked with Cd and Pb. They suggested that the improved growth was possibly due to a decreased metal uptake by the plant. Vivas et al. [22] reported that the inoculation of *Brevibacillus* sp. alleviated the toxicity of Zn in *Trifolium repens* by reducing the metal uptake by plants growing on a Zn contaminated soil. Inoculation with *Serratia* sp. MSMC541 decreased the metal translocation of *Lupinus luteus* when growing in a soil spiked with As, Cd, Pb and Zn [57]. They concluded that this strain protects the plants against metal toxicity by reducing their uptake and, in this way promoting plant growth. In our work, the weight of D19 plants increased in presence of *Pseudomonas* sp. 228 under Zn exposure, and after addition of *Pseudomonas* sp. 262 and *Serratia* sp. 246 in presence of Cd. In the case of the VR cultivar, the weight also increased in plants inoculated with *Serratia* sp. 246 in presence of Zn. The concentration of metals tended to decrease in roots of plants inoculated with *Pseudomonas* sp. 228 and *Serratia* sp. 246, although this decrease was only significant in the case of the D19 cultivar, after inoculation with *Pseudomonas* sp. 262 in presence of Cd. Taking this into account, our data support the hypothesis that bacteria have cultivar-dependent and metal-specific effects on plant growth. *Pseudomonas* sp. 262 is a promising endophyte because it can lower metal uptake in presence of Cd, decrease phytotoxicity, and improve plant growth.

### 2.2. Nutrient Status

In general, inoculation of bacterial strains did not have clear effects on the nutrient concentrations in both cultivars (Tables S1–S4). Macronutrients as Na and Ca were significantly lower in roots of, respectively, VR and D19 plants when plants were inoculated with *Serratia* sp. 246, *Pseudomonas* sp. 228 and 256 in presence of 1 mM of Zn (Table S1). In the case of exposure of the plants to 0.1 mM Cd, inoculation of *Arthrobacter* sp. 222, *Serratia* sp. 246, *Pseudomonas* sp. 228 and 262 led to lower K concentrations in shoots of the VR cultivar (Table S2).

Micronutrient concentrations changed in some cases after bacterial inoculation. When plants were grown in the presence of Zn, inoculation of *Serratia* sp. 246, *Pseudomonas* sp. 228 and 256 significantly decreased the Cu and Fe concentrations in roots of respectively the VR and D19 cultivars (Table S3). The concentrations of Cu in roots of Cd exposed plants of the VR cultivar were also lower after inoculation with *Arthrobacter* sp. 222, *Serratia* sp. 246 and *Pseudomonas* sp. 262 (Table S4). However, *Serratia* sp. 246 increased the Fe content in the shoots of the VR cultivar in presence of Zn (Table S3). The latter strain also increased the weight of VR plants exposed to Zn.

The lower Cu and Fe concentrations in roots of both cultivars when inoculated with *Serratia* sp. 246 and *Pseudomonas* (262 and 256) can be due to the above-mentioned bacterial mechanisms of metal sequestration and/or biosorption. Microorganisms indeed have developed complex mechanisms of metal resistance that can affect the availability of metals and nutrients [58,59]. PGPB can sequestrate elements through extracellular production of polysaccharides, by fixing elements such as Fe or Cu on the membrane or cell wall or they can precipitate them in the form of hydroxides or other insoluble metal salts [23,60,61]. Bacterial surfaces hold polar functional groups that can interact with cations [62]. In our work, the excess of Cd and Zn might induce the bacterial mechanisms of metal resistance that lower the availability of metals and also the solubility of other nutrients that might be precipitated on the cell surface.

The synthesis of siderophores is stimulated in presence of toxic metals in order to supply the appropriate amounts of ions to the plant and diminish the phytotoxicity symptoms [17,29]. This PGP characteristic plays an important role under soil conditions in which the nutrients are mainly present for the plants in unavailable chemical forms. Therefore, it can be expected that, under the growth conditions used in this study (sand moistened with half-strength Hoagland whether or not supplemented with Zn or Cd), the effects of the bacteria on nutrient uptake are less pronounced, since Fe is supplied in an appropriate concentration with the nutrient solution. Thus, the differences observed in the nutrient concentrations in the plants might also be due to the imbalance of nutrients generated by the presence of metals in the solution.

### 2.3. Lipid Peroxidation

TBA reactive compounds are produced as a result of peroxidation of membrane lipids. This process is initiated by excess of free radicals in consequence of oxidative stress. Increased levels of TBA reactive compounds are an indicator for physiological stress [63]. Many studies reported that levels of TBA reactive compounds increased in plants exposed to toxic concentrations of metals such as Cd, Zn, and Pb [64,65,66,67].

**Figure 2:**
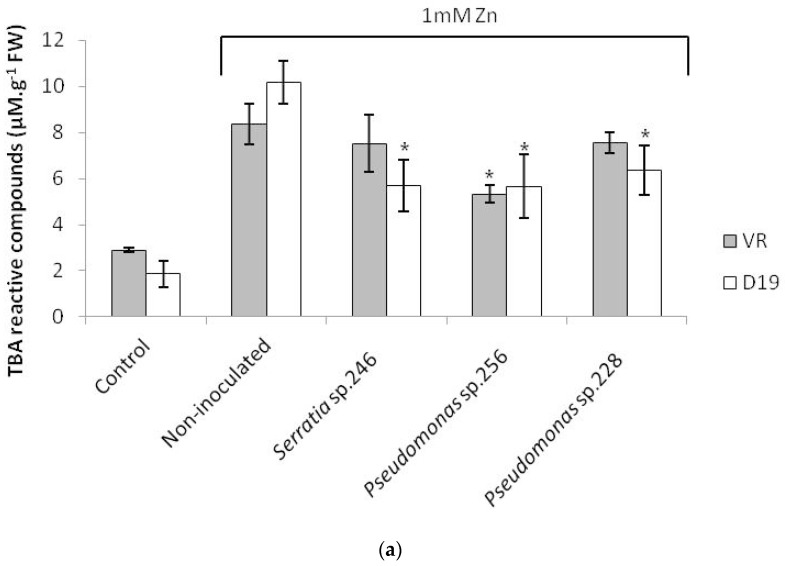

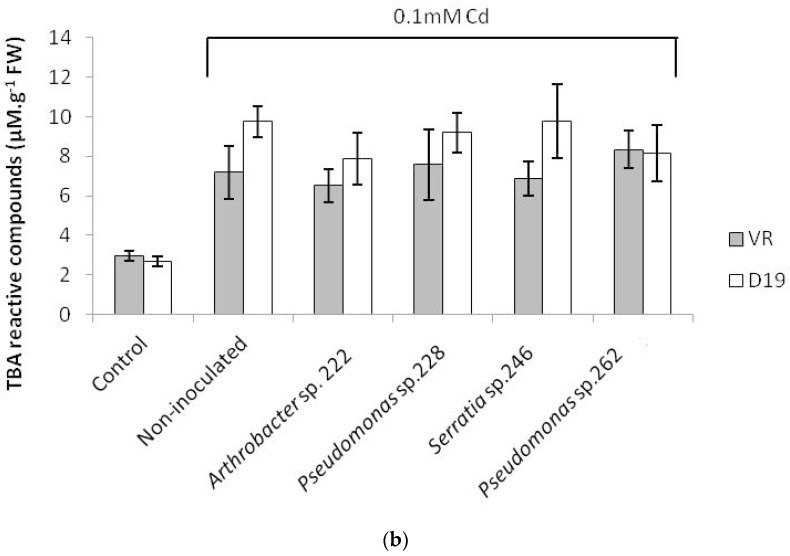
Thiobarbituric acid reactive compounds (µM·g^−1^ fresh weight) in roots of *H. tuberosus* cultivars VR and D19 after three weeks of exposure to: 1 mM of Zn (**a**); and 0.1 mM Cd (**b**). * Significant differences between inoculated and non-inoculated after Tukey’s test, *p* < 0.05; mean values ± SE; *n* = 4.

In the present work, exposure to 1 mM Zn and 0.1 mM Cd significantly increased the levels of TBA reactive compounds in roots of both cultivars of *H. tuberosus* (Figure 2). No significant differences in TBA-levels were found in leaves of metal-exposed plants compared to non-exposed plants. Nouairi et al. [68] obtained similar results for leaves of *Brassica juncea* exposed to 50 µM Cd. According to them, this result could be related with a tolerance mechanism of the plant to avoid oxidative stress generated by the presence of metals in the leaves. A reduction of the concentrations of TBA reactive compounds has been reported to result from increased activities of anti-oxidative enzymes, which limit H_2_O_2_ levels and membrane damage [69].

Interestingly, the inoculation of *Serratia* sp. 246, *Pseudomonas* sp. 256 and 228 significantly decreased the amounts of TBA reactive compounds in roots of the D19 cultivar grown in the presence of Zn (Figure 2a). The roots of the VR cultivar also contained lower levels of TBA reactive compounds when plants were inoculated with *Pseudomonas* sp. 228. In Cd exposed plants, no significant differences in TBA reactive compounds were observed between inoculated and non-inoculated plants (Figure 2b). Decreases of TBA reactive compounds after inoculation of PGPB were reported by several authors in different plant species. Pandey et al. [31] described that inoculation of *Ochrobactrum* strain CdSP9 lowered the content of TBA reactive compounds in hydroponically grown *Oryza sativa* exposed to Cd. Wan et al. [30] also observed that the inoculation of *Serratia nematodiphila* LRE07 decreased the concentration of TBA reactive compounds in *Solanum nigrum* exposed to Cd under hydroponic conditions.

These results suggest that the inoculated bacteria can assist metal exposed plants to keep the oxidative stress under control. In the inoculated plants, the Zn concentrations tended to decrease which could at least partially explain the lowering of TBA reactive compounds in inoculated plants.

### 2.4. Colonization of Enhanced Green Fluorescent Protein (EGFP): Tetracycline^®^ Pseudomonas sp. 262 in the Roots of H. tuberosus

*Pseudomonas* sp. 262 was able to grow in the presence of 0.8 mM Cd and showed in vitro the capacity to produce siderophores (in absence of iron), organic acids, indole acetic acid, acetoin and ACC deaminase (Table 1). Moreover, this bacterial strain increased the shoot weight of the D19 cultivar of *H. tuberosus* exposed to 0.1 mM Cd. Taking this into account, *Pseudomonas* sp. 262 was selected to be labeled with the EGFP: tetracycline^®^ plasmid to study the bacterial colonization of the roots of the *H. tuberosus* D19 cultivar.

**Figure 3:**
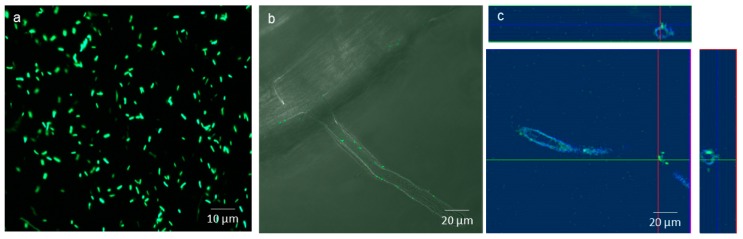
Confocal images of EGFP-labeled *Pseudomonas* sp. 262 colonising the root hairs of one-week-old seedlings of *H. tuberosus* D19 cultivar: (**a**) solution with EGFP-labeled *Pseudomonas* sp. strain 262 with blue light (488 nm) excitation; (**b**) single cells attached to a root hair; and (**c**) ortho-image of the root hair, showing bacterial cells (green) inside plant cells (in blue).

Figure 3a demonstrates that the conjugation was effective, since *Pseudomonas* sp. 262 showed fluorescence after blue light (488 nm) excitation, and was able to grow in presence of tetracycline (20 µg·mL^−1^). In Figure 3b, EGFP-*Pseudomonas* sp. 262 can be seen as single cells attached to the surfaces of root hairs. Two days after inoculation, bacterial cells were also found inside the root hair which can be observed from the orthogonal plot (Figure 3c). These results support that *Pseudomonas* sp. 262 is an endophytic strain.

Ma et al. [70] reported that another *Pseudomonas* sp. A3R3 isolated from roots of *Alyssum serpyllifolium* showed a high level of colonization in root and shoot interior of *Brassica juncea*. He et al. [27] also observed that *Rahnella* sp. JN6, originally isolated from *Polygonum pubescens*, could colonize the root, stem and leaf tissues of *Brassica napus*. *Rahnella aquatilis* SPb, an endophytic bacterial strain from *Ipomoea batatas* was inoculated in hybrid poplar and increased the growth of the cuttings in comparison with non-inoculated conditions, illustrating the beneficial effects of the strains in growth of another plant species not related with the initial host plant [71].

In our study, the EGFP-labeled bacterial strain was found in the root interior of *H. tuberosus* in the studied conditions. Since the inoculated bacterial cells were also found attached to the root hair surface, we suggest this one of the entry routes of the bacterial cells to the plant. Moreover, after inoculation of this strain, the growth of the *H. tuberosus* D19 cultivar improved significantly when exposed to Cd. This beneficial effect on plant growth, together with the visualization of the bacteria on the root hair surfaces and inside roots, indicates that a beneficial plant–microbe interaction was established.

## 3. Materials and Methods

### 3.1. Plant Material

Tubers of two cultivars of *H. tuberosus* (Violet de Rennes abbreviated as VR, and Blanc Précoce commonly named D19) were collected in spring in the field collection of IMIDRA (Instituto Madrileño de Investigación y Desarrollo Rural, Agrario y Alimentario; Madrid, Spain) to perform the experiments. The tubers were kept during two weeks at 4 °C for vernalization. After this period and before starting the experiments, the tubers were vigorously washed in tap water to remove the adhered soil.

### 3.2. PGPB Strains

Cultivable bacteria were isolated from soil, rhizosphere and plant-endosphere of *Brassica napus* growing on a Zn-contaminated site in Belgium [24]. Based on their PGP characteristics (Table 1), 3 Zn-tolerant strains (*Serratia* sp. strain 246, *Pseudomonas* sp. strain 228, and *Pseudomonas* sp. strain 256) and 4 Cd-tolerant strains (*Arthrobacter* sp. strain 222, *Pseudomonas* sp. strain 228, *Pseudomonas* sp. strain 262, and *Serratia* sp. strain 246) were selected to inoculate *H. tuberosus*. *Serratia* sp. strain 246 and *Pseudomonas* sp. strain 228 were inoculated in the presence of Zn and Cd because both strains showed high tolerance to grow with both metals. The strains were grown in 869 liquid medium [72] at 30 °C under shaking conditions.

### 3.3. Inoculation of PGPB Strains in H. tuberosus

Tuber slices with buds were incubated in 1 L plastic pots filled with moist quartz sand that were placed in a growth chamber at 25/12 °C, 14/12 h of photoperiod. The following conditions were established after one week of growth: (i) control plants grown in sand without metal and bacteria; (ii) non-inoculated, metal-exposed plants grown in the presence of metals (Cd or Zn), but without bacteria; (iii) plants inoculated with bacterial strains (*Arthrobacter* sp. 222, *Pseudomonas* sp. 228, 262 and *Serratia* sp. 246) and grown in presence of Cd; and (iv) plants inoculated with bacterial strains (*Pseudomonas* sp. 228, 256 and *Serratia* sp. 246) and grown in presence of Zn.

Half strength modified Hoagland’s solution (1 mM Ca (NO_3_)_2_·4H_2_O, 1.5 mM KNO_3_, 0.5 mM NH_4_H_2_PO_4_, 0.25 mM MgSO_4_·7H_2_O, 1 µM MnSO_4_·H_2_O, 12.5 µM H_3_BO_3_, 0.25 µM (NH_4_)_6_Mo_7_O_4_, 0.05 µM CuSO_4_·5H_2_O, 1 µM ZnSO_4_·7H_2_O, 10 µM NaFe^III^-EDTA, and demineralized water buffered with 1 mM of 2-(*N*-morpholino) ethanesulfonic acid, at pH 5.5 ± 0.5) was added to the sand until saturation. Metal exposures were performed by adding 0.1 mM of Cd (added as CdSO_4_·8H_2_O) or 1 mM of Zn (added as ZnSO_4_·7H_2_O) to the nutrient solution. Plants were watered every two days with the nutrient solution supplemented with metals. The metal concentrations used in this experiment were chosen based on a former study [73]. Two plants were put per pot with four independent replicates per treatment. The bacterial suspension (10^8^ cfu·mL^−1^) in buffer (10 mM MgSO_4_) was added into the pots. Buffer (10 mM MgSO_4_) without bacteria was added to the controls. After 3 weeks of growth, plants were harvested.

### 3.4. Plant Analysis

After harvest, the roots were rinsed in 10 mM sodium ethylenediaminetetraacetic acid (Na_2_EDTA) to remove the adhering metal-containing particles, and subsequently washed in distilled water. Plants were subdivided into leaves, stems and roots, weighed and dried in a forced air oven for 48 h at 60 °C to determine the dry weights. Subsequently, the dried tissues were individually ground and digested (30 mg) according to [47]. Total concentrations of metals and macro/micronutrients were determined by flame atomic absorption spectrometry (Fast Sequential Model AA240FS, Varian, Santa Clara, CA, USA). The quality of the digestion and analytical methods was verified by including blanks and certified reference materials (NCS DC73348 Brush Branches and Leaves, China National Analysis Center for Iron and Steel, and CTA-VTL-2 Virginia Tobacco Leaves, Polish Academy of Sciences and Institute of Nuclear Chemistry and Technology) with every set of samples. The recovery percentages for metals were: Cd (~95%) and Zn (~101%).

The membrane lipid peroxidation in the plant tissues was estimated in terms of the content of thiobarbituric acid reactive (TBA) compounds according to the method of [74], modified by [75]. The calibration curve was carried out with every set of samples, using 1,1,3,3-Tetraethoxypropane (TEP) as precursor of malondialdehyde (MDA). Absorbances were determined with a UV–Vis light spectrophotometer (Thermo Spectronic Helios Alpha, Thermo Fisher Scientific, Madison, WI, USA).

### 3.5. Evaluation of the Colonization Process: Localization of Inoculated EGFP Labeled Pseudomonas sp. 262

#### 3.5.1. Bacterial Strains and Growth Conditions

The receptor, *Pseudomonas* sp. 262 was grown in 284 minimal medium [76] supplemented with 0.4 mM of Cd (added as CdSO_4_·8H_2_O) at 30 °C. The donor, *Escherichia coli* strain dH5a, carrying the EGFP pMP4655 plasmid, was grown in 869 medium [72] supplemented with 20 µg·mL^−1^ tetracycline at 30 °C. The helper, *E. coli* strain dH5a, carrying the pRK2013 plasmid, was grown in 869 medium at 30 °C. Donor and helper were constructed in the Institute of Biology Leiden, Leiden University (The Netherlands) [39].

#### 3.5.2. Introduction of the EGFP: Tetracycline into *Pseudomonas* sp. 262

Triparental mating was carried out to label *Pseudomonas* sp. 262 with the EGFP: tetracycline® plasmid. The strains were grown in 869 medium at 30 °C under shaking conditions. Growth curves were obtained by diluting an overnight culture in order to verify the time needed to reach the appropriate optical density (OD) for conjugation (donor and helper OD 0.3–0.4, and receptor OD 0.7). The OD was measured at 660 nm every 30 min using a Visible Diode Array Spectrophotometer, Novaspec Plus, Amersham Biosciences, Piscataway, New York, United States. Once the appropriate OD was reached, the bacterial strains were centrifuged at 3000 rpm during 10 min, and then added to the mating filter in a Petri dish with 869 medium. After the conjugation, 284 minimal medium supplemented with 0.4 mM of Cd (added as CdSO_4_·8H_2_O) and tetracycline (20 µg·mL^−1^) was used to isolate the receptor labeled strains. Fluorescence of the strains was checked using a Nikon 80i fluorescence microscope (High-pressure Mercury Lamp; Excitation filters: 465–495 nm, dichroic mirror 505 nm, emission filter 515–555 nm. Objectives used: 40×/0.95 Air Plan Apo WD 0.14 mm and 100×/1.25 Oil Plan Apo WD 0.17 mm).

#### 3.5.3. Inoculation of EGFP *Pseudomonas* sp. 262 on Roots of *H. tuberosus*

Tuber slices with buds of *H. tuberosus* cultivar D19 were grown on coarse perlite moistened with a half strength modified Hoagland’s solution (see above) under greenhouse conditions (25–30 °C temperature and 70–90% relative humidity). The bacterial suspension (10^8^ cfu·mL^−1^) was added to the pots (0.2 L) after appearance of the first roots (at 5 days). Four repetitions were used. 

#### 3.5.4. Confocal Laser Scanning Microscopy

After 48 h of incubation, one-week-old plant roots were washed to remove weakly adhered bacterial cells and, subsequently, intact root preparations (at 25 °C) were observed with a Zeiss LSM510 confocal laser scanning microscope (Carl Zeiss, Jena, Germany) mounted on an Axiovert 200M. The objective used was 40×/1.1 water immersion (Zeiss LD C-Apochomat 40×/1.1 WKorr UV–VIS-IR, Carl Zeiss).

Excitation was performed at 488 nm using an Argon laser source. Backward GFP signal was filtered using a 500–550 nm band pass filter. Images were edited using the software Zen 2009 Light Edition (Carl Zeiss MicroImaging GmbH, Jena, Germany).

### 3.6. Statistical Analysis

Statistical analysis of data was performed using the IBM SPSS Statistics 19.0 software (Armonk, NY, USA). Two-way analysis of variance (ANOVA) and Tukey’s test were applied. Differences at *p* < 0.05 levels were considered significant. 

## 4. Conclusions

The effects of the bacterial strains on the growth of *H. tuberosus* differed in function of the metal, the inoculated bacterial strain and the plant cultivar. The improvement of growth and the decrease of the metal-induced stress were more pronounced in the D19 cultivar than in the VR cultivar. Three endophytes of *Brassica napus* enhanced the growth of the D19 cultivar exposed Cd or Zn. Only *Pseudomonas* sp. 228 increased Cd uptake. Using confocal microscopy, we observed that, two days after inoculation, EGFP-labeled *Pseudomonas* sp. 262 colonized the root surface and interior of *H. tuberosus*. In combination with the growth promotion that was observed after inoculation, this demonstrates an established plant–microbe interaction. Therefore, use of the D19 cultivar in combination with *Pseudomonas* sp. 228, *Serratia* sp. 246 and *Pseudomonas* sp. 262 holds promise for application in phytoremediation strategies on Cd-Zn contaminated soils. 

## Figures and Tables

**Table 1:** Metal tolerance and plant growth promoting (PGP) characteristics of selected bacterial strains for inoculation in *H. tuberosus* under hydroponic conditions with Cd and Zn, modified from [24].

Comp. ^1^	Strain	Identification	Accesion	Zn	Cd	Fe 0 µM	Fe 0.25 µM	OA	ACC	IAA	Ace	Psol	N fix
Soil	222	*Arthrobacter* sp.	KT461847	+++	+++	−	−	++	+++	−	−	−	+
Root	228	*Pseudomonas* sp.	KT461831	++	++	+	+	+	++	+	−	++	−
Root	246	*Serratia* sp.	KT461863	+++	+++	+	+	++	+++	++	−	−	−
Root	256	*Pseudomonas* sp.	KT461831	+++	+	+	+	-	+	++	+	+++	++
Root	262	*Pseudomonas* sp.	KT461831	+	+	++	−	+	+++	++	+	−	−

^1^ Compartment (Comp.), growth in the presence of Zn (1 mM) and Cd (0.8 mM), siderophores (Fe 0 µM and Fe 0.25 µM), Organic acids (OA), ACC (ACC deaminase activity), IAA (indole-3-acetic acid), Ace (Acetoin), phosphate solubilization (Psol), nitrogen fixation (N fix). + low, ++ medium, +++ high production, − absence of production.

**Table 2:** Total metal concentrations (mg·kg^−1^ dry matter) in two cultivars of *H. tuberosus* grown in absence (control) and in presence of 1 mM Zn or 0.1 mM Cd.

Treatments		VR		D19	
		Zn			
		Aerial	Root	Aerial	Root
Zn	Control	58 ± 14a	40 ± 10a	75 ± 23a	41 ± 5a
	Non-inoculated	1533 ± 149b	4533 ± 945c	1097 ± 175b	3862 ± 1063bc
	*Serratia* sp. 246	1155 ± 23b	4195 ± 355bc	1283 ± 207b	3455 ± 1767b
	*Pseudomonas* sp. 256	1349 ± 183b	4368 ± 442bc	1554 ± 299b	3484 ± 651b
	*Pseudomonas* sp. 228	975 ± 154b	2237 ± 368b	1317 ± 177b	3504 ± 1167b
		**Cd**			
Cd	Control	0.43 ± 0.09a	1.2 ± 0.2a	0.6 ± 0.1a	0.5 ± 0.2a
	Non-inoculated	152 ± 10c	1118 ± 177def	106 ± 44bc	889 ± 196cde
	*Arthrobacter* sp. 222	83 ± 6bc	492 ± 85bc	58 ± 8b	631 ± 140bc
	*Pseudomonas* sp. 228	112 ± 23bc	1250 ± 320ef	106 ± 18bc	1365 ± 145f
	*Serratia* sp. 246	24 ± 4b	908 ± 314cde	129 ± 45c	798 ± 65bcd
	*Pseudomonas* sp. 262	145 ± 29c	487 ± 57bc	81 ± 5bc	383 ± 107b

Different letters represent significant differences per column, cultivar and metal after Tukey’s test, *p* < 0.05; mean values ± SE; *n* = 4.

## Supplementary Materials

Supplementary materials can be found at www.mdpi.com/1422-0067/18/10/2026/s1.

## Abbreviations

PGPB	Plant Growth Promoting Bacteria
TBA	Thiobarbituric Acid
EGFP	Enhanced Green Fluorescent Protein
EDTA	Ethylenediaminetetraacetic Acid
MDA	Malondialdehyde
