# Supplementary material for: Cultivar and Metal-Specific Effects of Endophytic Bacteria in Helianthus tuberosus Exposed to Cd and Zn
Source: Int J Mol Sci. 2017 Sep 21;18(10):2026. doi: 10.3390/ijms18102026 (PMC5666708; doi:10.3390/ijms18102026)
Supplement: Supplementary file 1 [file ijms-18-02026-s001.pdf]

Table S1. Macro-nutrient concentration ($g \cdot 100g^{-1}$ dry matter) in aerial part and roots of two cultivars of *H. tuberosus* grown in absence (control) and presence of Zn (1 mM)

			**Zn (1mM)**			
		Control	Non-inoculated	*Serratia* sp. 246	*Pseudomonas* sp. 256	*Pseudomonas* sp. 228
**Aerial**						
VR	Ca	2.8 ± 0.5 c	1.8 ± 0.2 abc	1.6 ± 0.18 abc	1.5 ± 0.2 ab	1.7 ± 0.3 abc
	K	5.7 ± 0.8 b	4.1 ± 0.7 a	3.0 ± 0.5 a	2.8 ± 0.3 a	2.7 ± 0.4 a
	Mg	0.7 ± 0.1 c	0.52 ± 0.05 abc	0.49 ± 0.09 abc	0.40 ± 0.05 ab	0.45 ± 0.09 ab
	Na	0.09 ± 0.03 b	0.06 ± 0.01 a	0.09 ± 0.02 ab	0.09 ± 0.01 ab	0.09 ± 0.01 ab
D19	Ca	2.5 ± 0.5 bc	1.5 ± 0.3 ab	1.2 ± 0.4 a	1.3 ± 0.4 a	1.2 ± 0.3 a
	K	4.0 ± 0.5 b	2.9 ± 0.5 a	2.7 ± 0.4 a	2.7 ± 0.2 a	2.8 ± 0.19 a
	Mg	0.8 ± 0.1 bc	0.36 ± 0.09 a	0.33 ± 0.06 a	0.37 ± 0.07 a	0.42 ± 0.06 ab
	Na	0.09 ± 0.01 ab	0.08 ± 0.01 ab	0.07 ± 0.01 ab	0.04 ± 0.01 a	0.05 ± 0.01 a
**Root**						
VR	Ca	0.04 ± 0.01 a	0.6 ± 0.2 ab	0.22 ± 0.02 ab	0.30 ± 0.05 b	0.24 ± 0.04 ab
	K	1.5 ± 0.5 ns	1.8 ± 0.4	1.50 ± 0.07	1.4 ± 0.1	1.5 ± 0.1
	Mg	0.29 ± 0.04 ns	0.4 ± 0.07	0.15 ± 0.02	0.16 ± 0.03	0.15 ± 0.02
	Na	0.21 ± 0.04 ab	0.36 ± 0.02 b	0.10 ± 0.01 a	0.11 ± 0.01 a	0.12 ± 0.01 a
D19	Ca	0.11 ± 0.02 ab	0.58 ± 0.19 c	0.23 ± 0.03 ab	0.19 ± 0.05 ab	0.08 ± 0.04 a
	K	1.6 ± 0.5 ns	2.3 ± 0.5	1.3 ± 0.2	1.4 ± 0.1	1.9 ± 0.6
	Mg	0.3 ± 0.1 ns	0.13 ± 0.04	0.16 ± 0.02	0.15 ± 0.02	0.14 ± 0.05
	Na	0.16 ± 0.04 ab	0.16 ± 0.05 ab	0.12 ± 0.01 a	0.12 ± 0.02 a	0.09 ± 0.03 a

Different letters represent significant differences per row and cultivar, after Tukey's test, $p < 0.05$; mean values ± SE; n=4. ns: not significant.

Table S2. Macro-nutrient concentration (g·100g^{-1} dry matter) in aerial part and roots of two cultivars of *H. tuberosus* grown in absence (control) and presence of Cd (0.1mM).

			**Cd (0.1mM)**				
		Control	Non-inoculated	*Athrobacter* sp. 222	*Pseudomonas* sp. 228	*Serratia* sp. 246	*Pseudomonas* sp. 262
				**Aerial**			
VR	Ca	2.7 ± 0.1 d	1.9 ± 0.5 bc	1.2 ± 0.1 ab	0.9 ± 0.1 ab	0.6 ± 0.1 ab	1.1 ± 0.1 ab
	K	13 ± 1 d	12.9 ± 0.4 d	9 ± 1 bc	5 ± 1 a	3.8 ± 0.2 a	8 ± 1 b
	Mg	0.65 ± 0.04 cd	0.57 ± 0.04 abc	0.56 ± 0.06 abc	0.37 ± 0.07 a	0.44 ± 0.06 ab	0.60 ± 0.07 bcd
	Na	0.014 ± 0.003 a	0.031 ± 0.008 ab	0.05 ± 0.01 ab	0.017 ± 0.003 a	0.029 ± 0.008 a	0.05 ± 0.01 ab
D19	Ca	2.4 ± 0.6 cd	1.4 ± 0.5 abc	1.1 ± 0.3 ab	1.1 ± 0.1 ab	1.6 ± 0.2 abc	0.7 ± 0.1 a
	K	11 ± 1 cd	7 ± 2 ab	7 ± 2 bc	8 ± 1 b	8.2 ± 0.3 bc	5 ± 1 a
	Mg	0.79 ± 0.09 d	0.53 ± 0.09 abc	1.1 ± 0.1 abc	1.10 ± 0.02 abc	1.6 ± 0.06 cd	0.53 ± 0.09 abc
	Na	0.027 ± 0.007 ab	0.042 ± 0.009 ab	0.04 ± 0.01 bc	0.04 ± 0.01 ab	0.08 ± 0.02 c	0.032 ± 0.008 ab
				**Root**			
VR	Ca	0.38 ± 0.04 ab	0.4 ± 0.1 abc	0.29 ± 0.05 a	0.58 ± 0.03 bc	0.4 ± 0.1 abc	0.5 ± 0.1 c
	K	7 ± 1 a	5 ± 1 bc	3 ± 1 ab	5 ± 1 abc	4 ± 1 ab	3 ± 1 a
	Mg	0.29 ± 0.05 ab	0.17 ± 0.03 ab	0.17 ± 0.02 ab	0.24 ± 0.01 ab	0.17 ± 0.02 ab	0.23 ± 0.02 ab
	Na	0.06 ± 0.01 ab	0.1 ± 0.01 ab	0.16 ± 0.05 ab	0.08 ± 0.02 ab	0.072 ± 0.003 ab	0.09 ± 0.01 ab
D19	Ca	0.37 ± 0.04 ab	0.6 ± 0.2 bc	0.46 ± 0.03 abc	0.6 ± 0.1 bc	0.6 ± 0.1 bc	0.6 ± 0.1 bc
	K	4 ± 1 ab	4 ± 1 ab	3 ± 1 ab	3 ± 1 ab	3 ± 1 ab	2.5 ± 0.6 a
	Mg	0.15 ± 0.04 b	0.15 ± 0.03 a	0.15 ± 0.01 a	0.21 ± 0.05 ab	0.2 ± 0.02 ab	0.15 ± 0.03 a
	Na	0.15 ± 0.04 b	0.099 ± 0.004 ab	0.07 ± 0.01 a	0.09 ± 0.01 ab	0.09 ± 0.02 ab	0.08 ± 0.01 ab

Different letters represent significant differences per row and cultivar, after Tukey's test, $p < 0.05$; mean values ± SE; n=4. ns: not significant.

Table S3. Micro-nutrient concentration (mg.kg^{-1} dry matter) in aerial part and roots of two cultivars of *H. tuberosus* grown in absence (control) and presence of Zn (1 mM)

			Zn (1mM)			
		Control	Non-inoculated	*Serratia* sp. 246	*Pseudomonas* sp. 256	*Pseudomonas* sp. 228
**Aerial**						
VR	Cu	13.8 ± 1.6 abc	17.5 ± 1.3 c	14.1 ± 1.8 abc	14.1 ± 1.3 abc	17.3 ± 1.9 c
	Fe	194.1 ± 23.0 e	89.2 ± 12.6 bc	148.4 ± 13.7 d	130.5 ± 26.7 cd	82.8 ± 25.1 abc
	Mn	11.3 ± 1.7 abc	14.3 ± 2.8 abc	10.8 ± 0.7 ab	8.5 ± 1.0 a	16.6 ± 6.1 abc
D19	Cu	16.8 ± 2.8 bc	10.4 ± 1.4 a	10.6 ± 0.6 ab	13.4 ± 2.0 abc	10.4 ± 4.1 a
	Fe	145.1 ± 13.8 a	80.2 ± 11.2 abc	44.5 ± 13.6 ab	35.5 ± 6.8 a	67.3 ± 8.3 ab
	Mn	44.5 ± 4.0 d	15.7 ± 3.1 abc	16.7 ± 6.3 abc	21.1 ± 5.8 bc	22.6 ± 5.3 c
**Root**						
VR	Cu	17.3 ± 5.3 ab	18.9 ± 2.6 c	9.8 ± 0.3 ab	12.4 ± 2.6 b	11.4 ± 0.6 ab
	Fe	118.0 ± 36.4 ab	102.2 ± 18.9 ab	91.9 ± 7.8 ab	83.3 ± 5.9 ab	83.2 ± 8.1 ab
	Mn	2.2 ± 0.2 a	2.4 ± 0.1 ab	1.79 ± 0.05 ab	1.6 ± 0.1 a	1.8 ± 0.3 ab
D19	Cu	10.4 ± 1.5 ab	10.6 ± 3.0 ab	9.3 ± 3.0 ab	13.5 ± 2.6 b	6.8 ± 1.1 a
	Fe	141.5 ± 27.1 b	113.6 ± 21.7 b	71.5 ± 6.7 a	63.4 ± 6.7 a	62.4 ± 11.3 a
	Mn	6.1 ± 0.7 c	4.5 ± 1.3 bc	2.8 ± 0.2 ab	3.0 ± 0.5 b	3.3 ± 0.1 ab

Different letters represent significant differences per row and cultivar, after Tukey's test, $p < 0.05$; mean values ± SE; n=4

Table S4. Micro-nutrient concentration (mg.kg^{-1} dry matter) in aerial part and roots of two cultivars of *H. tuberosus* grown in absence (control) and presence of Cd (0.1mM).

			Cd (0.1mM)				
		Control	Non-inoculated	*Athrobacter* sp. 222	*Pseudomonas* sp. 228	*Serratia* sp. 246	*Pseudomonas* sp. 262
**Aerial**							
VR	Cu	7.4 ± 1.0 abc	8.0 ± 2.0 abc	3.0 ± 1.0 a	3.5 ± 0.3 ab	8.0 ± 0.9 abc	8.8 ± 1.3 bc
	Fe	65.9 ± 8.7 de	33.2 ± 10.3 abcd	11.6 ± 7.1 a	25.0 ± 4.7 abc	20.7 ± 7.0 ab	48.1 ± 9.0 abcd
	Mn	11.8 ± 1.1 c	3.2 ± 0.4 ab	2.4 ± 0.6 a	1.5 ± 0.4 a	1.5 ± 0.6 a	8.0 ± 2.1 b
D19	Cu	10.5 ± 2.3 c	9.4 ± 1.4 c	5.1 ± 0.8 abc	8.2 ± 1.6 abc	6.6 ± 1.2 abc	10.2 ± 3.3 c
	Fe	93.2 ± 16.9 e	48.7 ± 10.1 bcd	41.4 ± 7.7 abcd	32.0 ± 6.7 abcd	52.3 ± 12.8 cd	47.4 ± 9.4 bcd
	Mn	12.5 ± 2.1 c	3.8 ± 0.5 ab	1.3 ± 0.3 a	1.1 ± 0.3 a	2.6 ± 0.2 a	1.5 ± 0.4 a
**Root**							
VR	Cu	14.8 ± 1.3 bc	19.5 ± 4.3 c	4.4 ± 1.4 a	17.1 ± 2.6 bc	11.3 ± 5.8 ab	9.8 ± 0.8 ab
	Fe	193.8 ± 46.9 c	62.4 ± 13.7 b	63.2 ± 2.1 b	65.6 ± 13.2 b	60.0 ± 25.4 b	68.8 ± 16.4 b
	Mn	3.4 ± 0.9 c	0.9 ± 0.3 a	1.0 ± 0.2 ab	2.6 ± 0.6 abc	1.0 ± 0.1 ab	3.2 ± 0.7 bc
D19	Cu	10.2 ± 1.4 ab	10.3 ± 3.1 ab	5.5 ± 0.8 a	10.1 ± 3.1 ab	10.8 ± 1.6 ab	3.0 ± 1.1 a
	Fe	154.8 ± 49.6 c	34.3 ± 7.6 ab	47.3 ± 5.7 ab	68.7 ± 19.0 ab	52.7 ± 12.2 ab	10.1 ± 0.8 a
	Mn	2.7 ± 0.7 abc	1.5 ± 0.7 abc	1.9 ± 0.4 abc	2.2 ± 1.0 abc	0.5 ± 0.2 a	1.1 ± 0.4 ab

Different letters represent significant differences per row and cultivar, after Tukey's test, $p < 0.05$; mean values ± SE; n=4
